# Analysis of Lymphocytic DNA Damage in Early Multiple Sclerosis by Automated Gamma-H2AX and 53BP1 Foci Detection: A Case Control Study

**DOI:** 10.1371/journal.pone.0147968

**Published:** 2016-01-28

**Authors:** Ludwig Rasche, Lisa Heiserich, Janina Ruth Behrens, Klaus Lenz, Catherina Pfuhl, Katharina Wakonig, René Markus Gieß, Erik Freitag, Caroline Eberle, Jens Wuerfel, Jan Dörr, Peter Bauer, Judith Bellmann-Strobl, Friedemann Paul, Dirk Roggenbuck, Klemens Ruprecht

**Affiliations:** 1 NeuroCure Clinical Research Center, Charité - Universitätsmedizin Berlin, Berlin, Germany; 2 Clinical and Experimental Multiple Sclerosis Research Center, Charité - Universitätsmedizin Berlin, Berlin, Germany; 3 Department of Neurology, Charité - Universitätsmedizin Berlin, Berlin, Germany; 4 Medipan GmbH, Berlin-Dahlewitz, Germany; 5 Department of Medical Biometrics and Clinical Epidemiology, Charité - Universitätsmedizin Berlin, Berlin, Germany; 6 MIAC AG, Basel, Switzerland; 7 Experimental and Clinical Research Center, Charité - Universitätsmedizin Berlin and Max Delbrück Center for Molecular Medicine, Berlin-Buch, Germany; 8 Institute of Neuroradiology, Universitätsmedizin Göttingen, Göttingen, Germany; 9 Faculty of Science, Brandenburg University of Technology Cottbus - Senftenberg, Senftenberg, Germany; Medical University of Innsbruck, AUSTRIA

## Abstract

**Background:**

In response to DNA double-strand breaks, the histone protein H2AX becomes phosphorylated at its C-terminal serine 139 residue, referred to as γ-H2AX. Formation of γ-H2AX foci is associated with recruitment of p53-binding protein 1 (53BP1), a regulator of the cellular response to DNA double-strand breaks. γ-H2AX expression in peripheral blood mononuclear cells (PBMCs) was recently proposed as a diagnostic and disease activity marker for multiple sclerosis (MS).

**Objective:**

To evaluate the significance of γ-H2AX and 53BP1 foci in PBMCs as diagnostic and disease activity markers in patients with clinically isolated syndrome (CIS) and early relapsing-remitting MS (RRMS) using automated γ-H2AX and 53BP1 foci detection.

**Methods:**

Immunocytochemistry was performed on freshly isolated PBMCs of patients with CIS/early RRMS (n = 25) and healthy controls (n = 27) with γ-H2AX and 53BP1 specific antibodies. Nuclear γ-H2AX and 53BP1 foci were determined using a fully automated reading system, assessing the numbers of γ-H2AX and 53BP1 foci per total number of cells and the percentage of cells with foci. Patients underwent contrast enhanced 3 Tesla magnetic resonance imaging (MRI) and clinical examination including expanded disability status scale (EDSS) score. γ-H2AX and 53BP1 were also compared in previously frozen PBMCs of each 10 CIS/early RRMS patients with and without contrast enhancing lesions (CEL) and 10 healthy controls.

**Results:**

The median (range) number of γ-H2AX (0.04 [0–0.5]) and 53BP1 (0.005 [0–0.2]) foci per cell in freshly isolated PBMCs across all study participants was low and similar to previously reported values of healthy individuals. For both, γ-H2AX and 53BP1, the cellular focus number as well as the percentage of positive cells did not differ between patients with CIS/RRMS and healthy controls. γ-H2AX and 53BP1 levels neither correlated with number nor volume of T2-weighted lesions on MRI, nor with the EDSS. Although γ-H2AX, but not 53BP1, levels were higher in previously frozen PBMCs of patients with than without CEL, γ-H2AX values of both groups overlapped and γ-H2AX did not correlate with the number or volume of CEL.

**Conclusion:**

γ-H2AX and 53BP1 foci do not seem to be promising diagnostic or disease activity biomarkers in patients with early MS. Lymphocytic DNA double-strand breaks are unlikely to play a major role in the pathophysiology of MS.

## Introduction

Multiple sclerosis (MS) is a chronic inflammatory demyelinating and neurodegenerative disease of the central nervous system and the leading cause for permanent neurological disability in young adults [1]. The diagnosis of MS can be challenging, in particular in the early phase of the disease [2]http://www.ncbi.nlm.nih.gov/pubmed/18805839. Furthermore, only a proportion of MS lesions seen on conventional magnetic resonance imaging (MRI) is associated with overt clinical symptoms, complicating clinical assessment of disease activity [3,4]. Finally, the course of MS is highly variable as is the response to immunomodulatory therapies [5]. Thus, there is an immanent need for reliable diagnostic, disease activity, prognostic, and therapy response markers in patients with MS [6]. Numerous blood-based biomarkers have therefore been evaluated in MS, though very few have entered clinical practice [7]

In response to DNA double-strand breaks the histone protein H2AX becomes phosphorylated at its serine 139 residue in the vicinity of the DNA break site [8]. The thus phosphorylated H2AX is termed γ-H2AX and plays a central role in the cellular DNA double-strand break response pathway by providing a platform for the recruitment of other DNA damage response and repair factors [9,10]. Among those, one key element is p53-binding protein 1 (53BP1), which, much like γ-H2AX, accumulates in discrete nuclear foci at DNA double-strand break sites and has important regulatory functions for the cellular response to DNA double-strand breaks [11,12]. Nuclear γ-H2AX and 53BP1 foci can be visualized in isolated peripheral blood mononuclear cells (PBMCs) by immunofluorescence microscopy. Indeed, digital fluorescence microscopy employing novel pattern recognition algorithms has recently been established for the automated analysis of DNA damage response foci, paving the way for a much needed standardization in this field [13–16].

A recent pilot study suggested γ-H2AX expression in PBMCs as a possible diagnostic as well as disease activity marker for relapsing remitting MS (RRMS), as patients with RRMS exhibited higher levels of γ-H2AX positive cells than healthy controls and levels of γ-H2AX positive cells were associated with MRI measures of disease activity in patients with RRMS [17]. Oxidative stress has been implicated in the pathogenesis of MS [18,19]. Because elevated levels of reactive oxygen species may cause DNA damage, a possible link between DNA damage markers in PBMCs and MS may be hypothesized [17].

To further scrutinize the possible association of lymphocytic DNA double-strand break markers and MS, we herein analyzed nuclear γ-H2AX and 53BP1 foci in freshly isolated PBMCs from 25 patients with clinically isolated syndrome (CIS) or early RRMS and 27 healthy controls as well as in previously frozen PBMCs from 20 Patients with CIS or early RRMS and 10 healthy controls using fully automated immunofluorescence microscopy [14,15]. Moreover, we correlated γ-H2AX and 53BP1 levels with MRI measures of disease activity.

## Patients and Methods

### Patients and healthy controls

The study was approved by the institutional review board of Charité–Universitätsmedizin Berlin (EA1/182/10). All participants provided written informed consent.

PBMCs were freshly isolated from blood samples obtained by peripheral venipuncture from 25 patients with CIS or early RRMS and 27 age and sex matched healthy controls between March and July 2014. Additionally, previously frozen PBMC samples, obtained between April 2012 and July 2014, from each 10 age and sex matched patients with CIS/early RRMS with and without contrast enhancing lesions (CEL) and 10 age and sex matched healthy controls were included in the study. All patients were participating in an ongoing prospective observational study of patients with early MS (Berlin CIS Cohort; NCT01371071), which started recruitment in January 2011. Inclusion criteria were: age>18 years, a first clinical event suggestive of inflammatory demyelination (i.e. CIS) not meeting the McDonald 2010 criteria for RRMS [20] within six months before inclusion into the study or a diagnosis of RRMS according to the McDonald 2010 criteria within 24 months before inclusion into the study. Exclusion criteria were: a history of alcohol or drug abuse, any conditions precluding MRI examinations and any ocular diseases precluding optical coherence tomography. The patients investigated in the present analysis had not been treated with corticosteroids for at least two months prior to inclusion into the study. At the time of blood withdrawal, patients underwent a thorough neurological examination, including determination of the expanded disability status scale (EDSS) score.

### PBMC isolation

**Fig 1:**
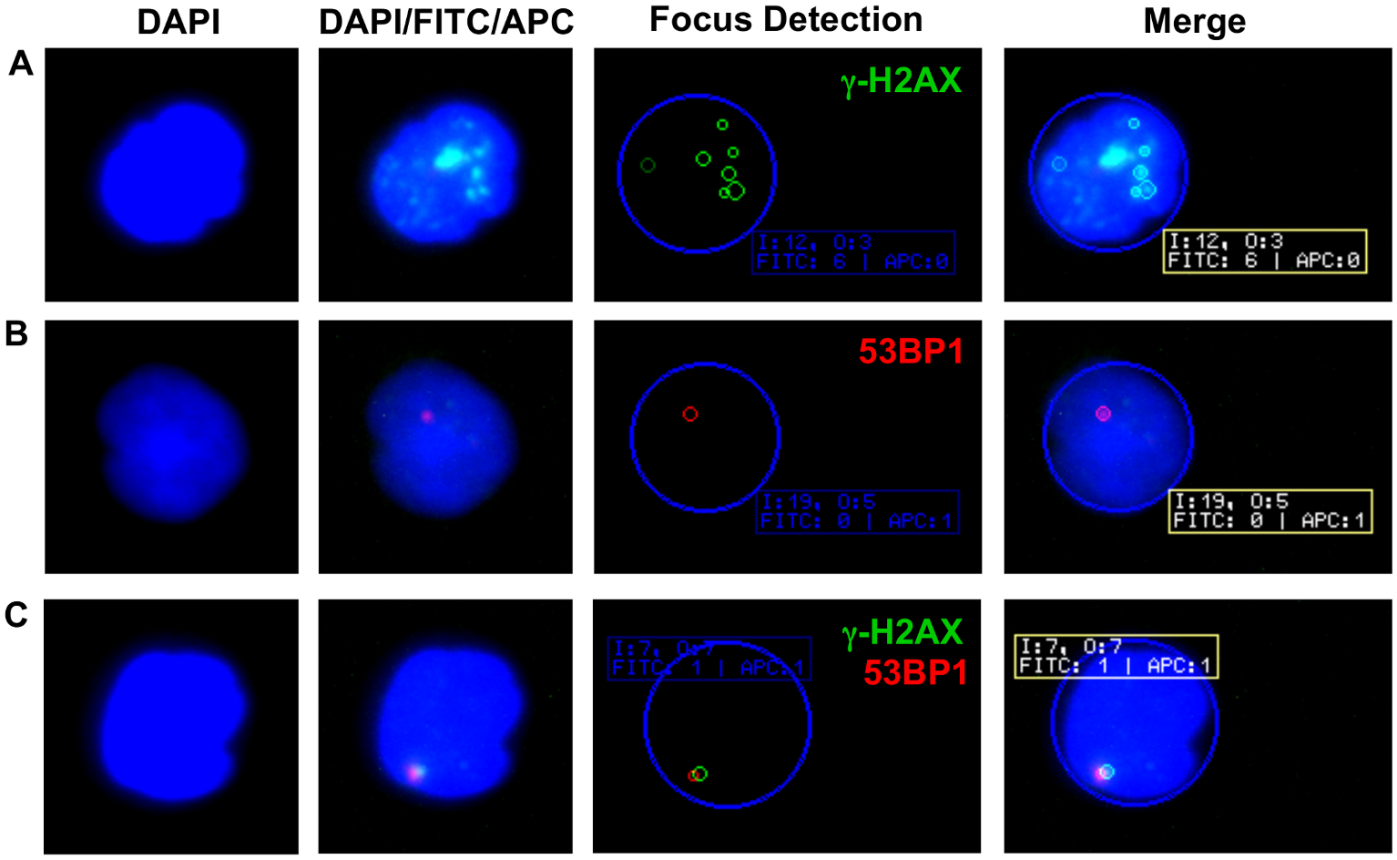
Examples of γ-H2AX and 53BP1 foci in PBMCs. (A) Multiple nuclear γ-H2AX foci (green) are clearly discernible in a lymphocyte of a healthy control. (B) Single 53BP1 focus (red) in a lymphocyte nucleus of a patient with RRMS. (C) Colocalization of nuclear γ-H2AX and 53BP1 foci in a lymphocyte of a healthy control. In the automated focus detection, the blue circle represents the DAPI stained nucleus. Small green and red circles highlight γ-H2AX and 53BP1 foci as automatically detected by the AKLIDES^®^ reading system. The system assigns distinct image (I) and object (O) numbers and indicates the number of foci detected in each fluorescence channel (FITC or APC). Note the difference in highlighted (n = 7) and counted (n = 6) γ-H2AX foci in (A), which is explained by a weaker focus fluorescence intensity not meeting the preset definitions for scoring of γ-H2AX foci.

**Fig 2:**
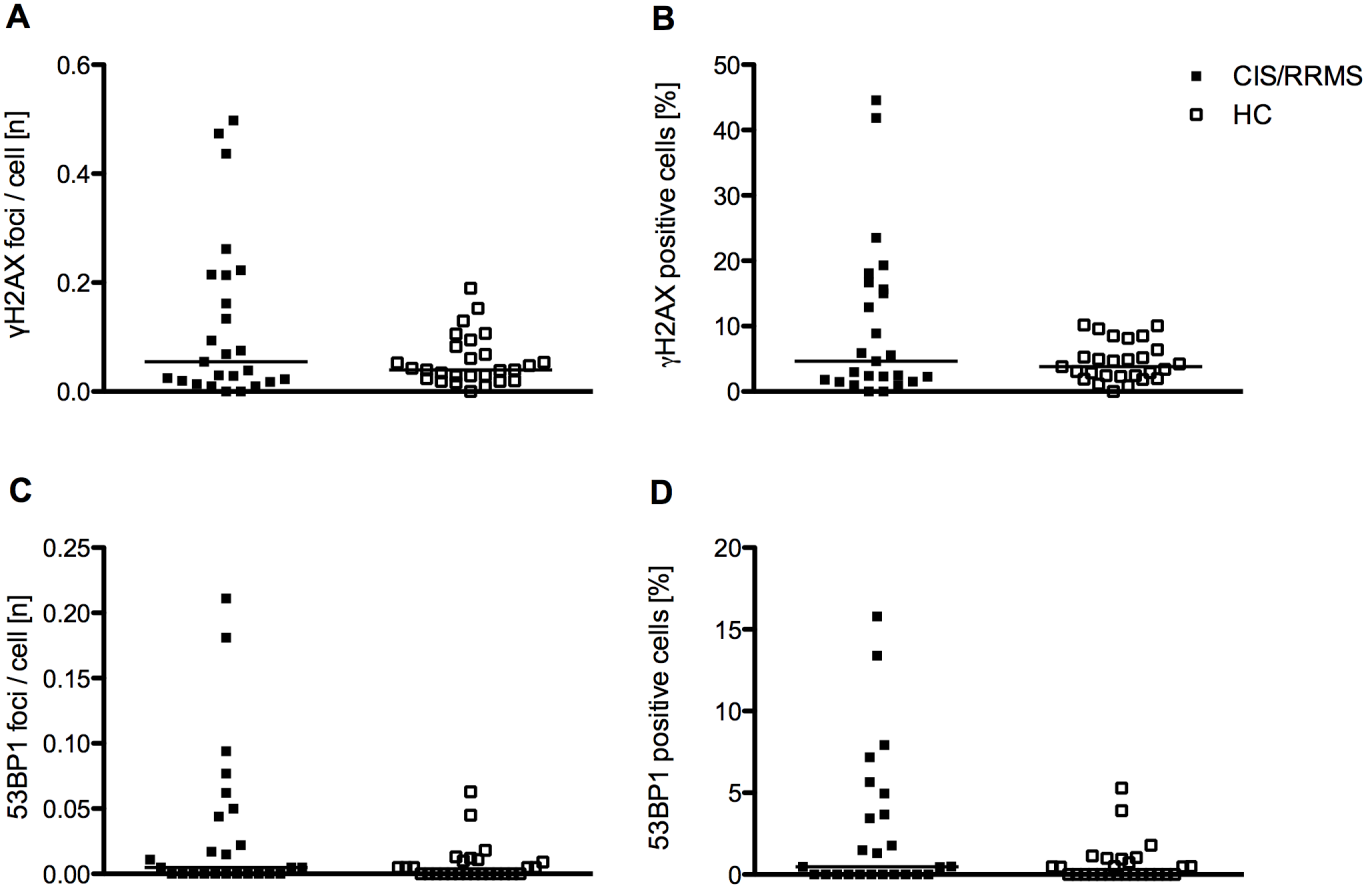
γ-H2AX and 53BP1 levels in freshly isolated PBMCs of patients and healthy controls. (A) γ-H2AX foci per cell in patients with CIS/early RRMS and healthy controls. (B) Percentage of γ-H2AX positive cells in patients with CIS/early RRMS and healthy controls. (C) 53BP1 foci per cell in patients with CIS/early RRMS and healthy controls. (D) Percentage of 53BP1 positive cells in patients with CIS/early RRMS and healthy controls. Each data point represents the median of n = 6 separate measurements per individual. In each of the six separate measurements approximately n = 100 cells were scored. The horizontal bar indicates the median.

**Fig 3:**
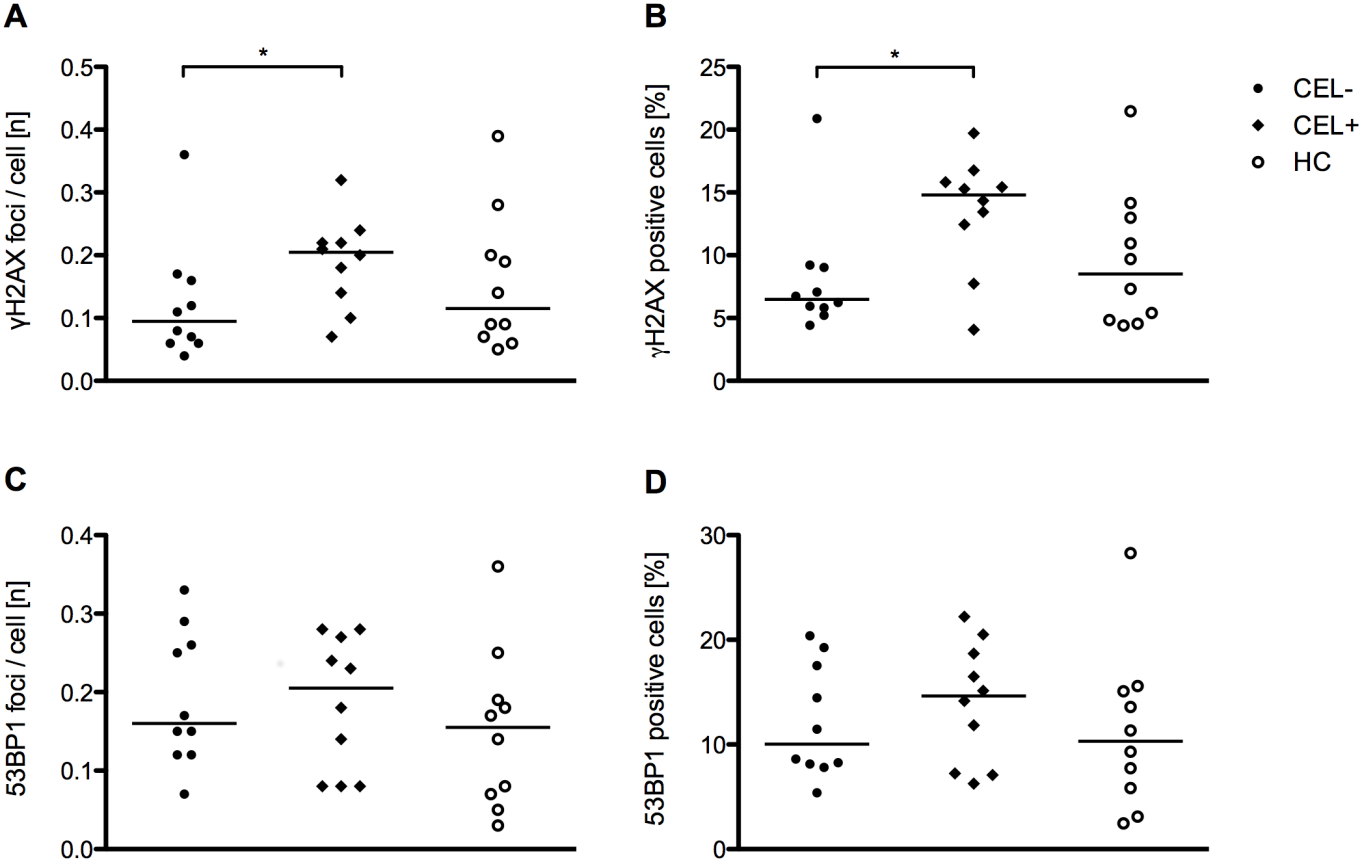
γ-H2AX and 53BP1 in frozen PBMCs of CIS/early RRMS patients with and without CEL and healthy controls. (A) γ-H2AX foci per cell in patients with CIS/early RRMS without CEL (CEL-; n = 10), with CEL (CEL+; n = 10) and healthy controls (HC; n = 10). (B) Percentage of γ-H2AX positive cells in patients with CIS/early RRMS without CEL, with CEL and healthy controls. (C) 53BP1 foci per cell in patients with CIS/early RRMS without CEL, with CEL and healthy controls. (D) Percentage of 53BP1 positive cells in patients with CIS/early RRMS without CEL, with CEL and healthy controls. The horizontal bar indicates the median. *p<0.05, Mann Whitney test.

PBMCs were isolated from heparinized blood (27 ml) within 4.5 hours of blood withdrawal through Biocoll (Biochrom GmbH, Berlin, Germany) density centrifugation at 760 g for 20 minutes at room temperature. The lymphocyte/monocyte fraction was recovered and washed in phosphate buffered saline (PBS, Biochrom GmbH, Berlin, Germany) at 560 g for 20 minutes and at 400 g for 15 minutes. Cell pellets were resuspended in PBS at a concentration of 1x10^6^ cells/ml. Experiments shown in Figs 1 and 2 were performed with freshly isolated PBMCs. Experiments shown in Fig 3 were performed with previously frozen PBMCs. Freezing and thawing of PBMCs were carried out according to established protocols. PBMCs were frozen in RPMI-1640 (Gibco, Thermo Fisher Scientific Inc., Waltham. USA) containing 10% dimethyl sulfoxide (Sigma Aldrich Chemie GmbH, Munich, Germany), 20% fetal bovine serum (Biochrom GmbH, Berlin, Germany) and 1% Hepes (Gibco) and stored in liquid nitrogen. For thawing, aliquots were removed from liquid nitrogen and thawed at 37°C for 1 minute. Cells were quickly transferred into 10 ml of wash medium (5% fetal bovine serum, 1% Hepes and 94% RPMI-1640) at room temperature and washed two times at 300 g for 5 minutes before resuspension in PBS at a concentration of 1x10^6^ cells/ml.

### Microscope Slide preparation

PBMCs were transferred to 6 well Teflon coated microscope slides (Tekdon Inc., Myakka City, United States) at a concentration of 5x10^4^ cells per well and incubated for 10 minutes at room temperature. Cells were subsequently fixed with 2% paraformaldehyde (Merck Chemicals GmbH, Schwalbach, Germany) for 15 minutes at room temperature. Slides were washed in PBS for 30 minutes and stored in dry conditions at 4°C until further processing.

### Immunocytochemistry

Immunocytochemistry was performed following standard operating procedures at Medipan GmbH, Berlin-Dahlewitz, Germany. Fixed cells on the microscope slide were permeabilized with Triton X 100 (0.2% in 1% bovine serum albumin [BSA]/PBS) (BioUltra, Sigma-Aldrich Chemie GmbH, Taufkirchen, Germany) for five minutes at 4°C and subsequently washed three times in 1% BSA/PBS at room temperature. For co-immunostaining, slides were simultaneously incubated with a mouse monoclonal IgG antibody specifically detecting H2AX phosphorylated at serine 139 (Merck Millipore, Billerica, USA) and with anti-53BP1 rabbit polyclonal IgG (Novus Biologicals, Cambridge, United Kingdom) at a dilution of 1:200 in 1% BSA/PBS for one hour. After three further wash steps in PBS for ten minutes, secondary antibodies, Alexa Fluor 488 goat anti-mouse IgG (detected in the FITC channel; Invitrogen GmbH, Karlsruhe, Germany) and Alexa Fluor 647 goat anti-rabbit IgG H&L (detected in the APC channel; Invitrogen GmbH, Karlsruhe, Germany), were added simultaneously in a dilution of 1:500 in 1% BSA/PBS for one hour at room temperature. Slides were washed again three times in PBS for ten minutes and mounted using a 4,6´-diamidino-2-phenylindole (DAPI) containing mounting medium (GA Generic Assays GmbH, Berlin-Dahlewitz, Germany) for DNA counterstaining.

### Automated analysis of γ-H2AX and 53BP1 foci

Enumeration of γ-H2AX and 53BP1 foci was carried out as previously described using the automated AKLIDES^®^ reading system (Medipan GmbH, Berlin-Dahlewitz, Germany) [14–16]. The system uses a motorized inverse fluorescence microscope (Olympus IX81, Olympus, Hamburg, Germany) with a motorized scanning stage and is controlled by software using a set of mathematical algorithms to detect and evaluate immunofluorescence patterns. For each sample approximately 100 cells on each well were selected randomly for analysis. According to our presets, only convex nuclei with diameters between 2–15 μm were evaluated. This way, monocytes, granulocytes, heavily damaged cells, and cell aggregates were excluded. As outlined before, both cells with a high amount of induced DNA double-strand breaks as well as early apoptotic cells can show a pan-nuclear staining [21–23]. However, γ-H2AX foci analysis by the automated interpretation system AKLIDES^®^ cannot distinguish between these two instances. Thus, to avoid confounding of apoptotic cells and cells with a high amount of induced DNA double-strand breaks, cells with an γ-H2AX signal comprising ≥70% of the DAPI signal were excluded from the analysis [23]. The following exposure times were used for the different channels: DAPI default exposure 24 ms, FITC default exposure 1000 ms, APC default exposure 1000 ms. Sub-nuclear foci had to meet the following requirements to be counted as such: minimum diameter 0.2 μm, maximum diameter 1.2 μm, minimum intensity 70 gray values on a 8 bit gray scale (0–255 with 0 being black) above background intensity. Results were expressed as the quotient of the γ-H2AX or 53BP1 focus number divided by the total number of assessed cells (cellular focus number) and as the percentage of cells carrying foci among all assessed cells (percentage of positive cells). The median values from separate measurements of six separate wells containing PBMCs from one individual were used for further statistical analysis. In setup experiments, PBMC samples were split and either processed immediately or after 2.5 and 4.5 hours. Compared to immediately processed cells, no significant time dependent change of γ-H2AX and 53BP1 foci could be observed 2.5 and 4.5 hours after blood withdrawal, arguing against a possible confounding influence of time between blood withdrawal and PBMCs processing on γ-H2AX and 53BP1 expression (data not shown).

### Magnetic resonance imaging

Three-dimensional whole brain MR data (1mm^3^) were acquired on a 3 Tesla (3T) whole-body MRI (Magnetom Trio with TIM, Siemens Healthcare AG, Erlangen, Germany), using a clinical routine 12-channel head coil. For T2-weighted imaging (T2w), a single slab three-dimensional T2w turbo-spin-echo (TSE) sequence with high sampling efficiency (SPACE) was selected (TE 388 ms, TR 6000 ms, flip angle 120°) as well as a three-dimensional fluid attenuated inversion recovery sequence (SPACE-FLAIR; TE 502 ms, TR 5000 ms, TI 2100 ms, flip angle 120°), followed by an axial two-dimensional double-echo proton density/T2w sequence (TE 14/87 ms, TR 3400 ms, flip angle 120°, voxel resolution 1 x 1 x 3 mm^3^, no gap). Contrast enhanced images were acquired by a volumetric interpolated brain examination sequence optimized for short acquisition time with asymmetric k-space sampling and interpolation (VIBE; 1mm^3^, TE 2.2 ms, TR 4.85 ms, flip 9°) 8 min after weight adjusted 0.1 mM Gadubutrol (Bayer Vital GmbH, Leverkusen, Germany) injection. T2w lesions as well as CEL were counted. T2w as well as CEL lesion volume was calculated using the OsiriX software toolbox (OsiriX foundation, Geneva, Switzerland) and in-house applications. Experienced MRI raters were blinded to the results of any laboratory studies.

### Statistics

Significance of different frequencies was assessed by 2 × 2 or 2 × 3 Fisher´s exact test. Significance of differences in γ-H2AX and 53BP1 cellular focus numbers and percentage of positive cells was assessed by Mann Whitney test. Three-group comparisons were performed by Kruskall-Wallis test. Correlation analysis between DNA damage parameters, clinical data, and MRI parameters was carried out by Spearman correlation. All statistical tests were performed with GraphPad Prism Version 5.0d. *P*-values <0.05 were considered significant.

## Results

### Participants

Demographics, clinical and MRI data, as well as immunomodulatory treatments of the 25 patients with CIS (n = 12) or RRMS (n = 13) and the 27 healthy controls of which freshly isolated PBMC were analyzed in this study are summarized in Table 1. The sex (*p* = 0.36) and age distribution (*p* = 0.93) did not differ between the groups of patients and healthy controls.

**Table 1:** Demographic and clinical data of patients and healthy controls of which freshly isolated PBMCs were analyzed in this study.

	Patients	Healthy Controls	*P*-value
Number	25	27	n/a
CIS/early RRMS (number)	12/13	n/a	n/a
Female/Male (number)	16/9	21/6	*p* = 0.36
Age, years, median (range)	33 (21–52)	29 (20–56)	*p* = 0.93
EDSS, median (range)	1.5 (0–5.5)	n/a	n/a
T2w-lesions, number, median (range)	5 (0–107)	n/a	n/a
CEL, number, median (range)	0 (0–1)	n/a	n/a
Immunomodulatory treatment, number			
None	15	n/a	n/a
Glatiramer acetate	5	n/a	n/a
Interferon-beta	3	n/a	n/a
Dimethyl fumarate	1	n/a	n/a
Fingolimod	1	n/a	n/a

CIS = clinically isolated syndrome, RRMS = relapsing remitting multiple sclerosis, EDSS = expanded disability status scale, T2w = T2 weighted, CEL = contrast enhancing lesions, n/a = not applicable

### Levels of γH2AX and 53BP1 in freshly isolated PBMCs do not differ in patients with CIS/early RRMS and healthy controls

To compare the numbers of lymphocytic γ-H2AX and 53BP1 foci in freshly isolated PBMCs from patients with CIS/early RRMS and healthy controls, we used a previously established immunocytochemical staining technique combined with an automated reading system [14,15]. With this methodology, γ-H2AX and 53BP1 foci could be visualized as clearly discernible intranuclear dots, examples of which, as well as of the automated detection of γ-H2AX and 53BP1 foci, are shown in Fig 1.

Intranuclear γ-H2AX foci were detectable in PBMCs of 23 out of 25 (92%) patients with CIS/early RRMS and 26 out of 27 (96%) healthy controls (*p* = 0.6). While 53BP1 foci were overall less frequently detectable than γ-H2AX foci, the detection frequency of 53BP1 foci did neither differ between patients (14 out of 25; 56%) and controls (13 out of 27; 48%; *p* = 0.6). The median (range) number of γ-H2AX foci per cell across all study subjects was low (0.04 [0–0.49]), as was the median frequency of 53BP1 foci per cell across all study subjects (0.005 [0–0.21]). The median (range) percentage of positive cells across all study subjects for γ-H2AX was 4.02% (0–44.57) and for 53BP1 0.46% (0–15.79). Taken all study subjects together, there was a strong correlation of γ-H2AX and 53BP1 foci numbers per cell (r = 0.60; *p*<0.0001) as well as of the percentage of γ-H2AX and 53BP1 positive cells (r = 0.62; *p*<0.0001).

The number of γ-H2AX foci per cell did not differ (*p* = 0.49) between patients with CIS/early RRMS and healthy controls (Fig 2A). Likewise, the percentage of cells positive for γ-H2AX did not differ between patients and healthy controls (*p* = 0.49) (Fig 2B). Similarly, there was no significant difference of 53BP1 focus numbers (*p* = 0.18) and of the percentage of 53BP1 positive cells between patients and healthy controls (*p* = 0.17) (Fig 2C and 2D). Furthermore, γ-H2AX focus numbers (*p* = 0.11) and the percentage of γ-H2AX positive cells (*p* = 0.12) were not different in patients with CIS compared to patients with early RRMS. A comparison of patients treated with immunomodulatory therapies (n = 10) and untreated patients (n = 15) revealed no significant difference of γ-H2AX focus numbers per cell or the percentage of γ-H2AX positive cells (*p* = 0.11 for both) and no significant difference for 53BP1 focus numbers per cell or the percentage of positive cells (*p* = 0.17 for both). Likewise, a subgroup analysis of smokers (n = 7) and non-smokers (n = 18) within the group of patients showed no significant difference of γ-H2AX focus numbers per cell (*p* = 0.62) or of the percentage of γ-H2AX positive cells (*p* = 0.54) and no significant difference of 53BP1 focus numbers per cell (*p* = 0.88) or of the percentage of 53BP1 positive cells (*p* = 0.92). Finally, neither in the groups of patients nor of healthy controls was there a correlation of age and γ-H2AX or 53BP1 focus numbers per cell or of the percentage of γ-H2AX or 53BP1 positive cells.

### Levels of γ-H2AX and 53BP1 in freshly isolated PBMCs do not appear to be promising disease activity markers in patients with CIS/early RRMS

We next asked whether levels of γ-H2AX and 53BP1 in freshly isolated PBMCs from patients with CIS/early RRMS might be associated with radiological or clinical measures of MS. We therefore correlated the number and volume of T2w lesions as well as the number and volume of CEL on MRI performed at the time of blood withdrawal with levels of γ-H2AX and 53BP1. In the vast majority of patients (23/25) MRI was performed on the day of blood withdrawal. The delay between blood withdrawal and MRI in the remaining 2 patients was 5 and 6 days. γ-H2AX focus numbers per cell and the percentage of γ-H2AX positive cells did not correlate with T2w lesion load (r = 0.15; *p* = 0.49 and r = 0.13; *p* = 0.54) or T2w lesion volume (r = 0.14; *p* = 0.45 and r = 0.13; *p* = 0.50). Likewise, 53BP1 focus numbers per cell and the percentage of 53BP1 positive cells did not correlate with T2w lesion load (r = 0.30; *p* = 0.13 and r = 0.33; *p* = 0.13) and T2w lesion volume (r = 0.33; *p* = 0.08 and r = 0.38; *p* = 0.05). Only two patients had each one CEL. Although one of these patients had relatively high γ-H2AX (0.46 foci/cell; 41.9% positive cells) and 53BP1 levels (0.20 foci/cell; 15.8% positive cells), the other patient had low γ-H2AX (0.04 foci/cell; 3% positive cells) and 53BP1 levels (0.005 foci/cell, 0.5% positive cells). Clinical disability as measured by the EDSS did not correlate with γ-H2AX focus numbers per cell (r = 0.20, *p* = 0.32) or percentage of γ-H2AX positive cells (r = 0.20, *p* = 0.33). Likewise, there was no correlation of the EDSS and 53BP1 focus numbers per cell (r = 0.31; p = 0.12) or the percentage of 53BP1 positive cells (r = 0.30; *p* = 0.14).

As seen in Fig 2A, there were three individuals among the patient group with relatively high γ-H2AX foci numbers per cell. A detailed analysis of these three outliers showed no major common characteristics. One of these patients (age: 33 years, sex: female, EDSS: 1.5) showed a high T2w lesion count (n = 107), one CEL and was treated with fingolimod. One patient (age: 44 years, sex: male, EDSS: 5.5) had a moderate T2w lesion load (n = 13), no CEL, and was treated with glatiramer acetate. The third patient (age: 35, sex: male, EDSS: 0) had one T2w lesion without corresponding contrast enhancement and did not receive any immunomodulatory therapy.

### Analysis of γ-H2AX and 53BP1 in frozen PBMCs from patients with CIS/early RRMS with and without CEL and healthy controls

Because of the small number of freshly isolated PBMC samples from patients with CIS/early RRMS with CEL, we aimed to increase the number of patients with CEL. As for logistic reasons we were unable to obtain fresh PBMCs from a higher number of patients with CEL, we used previously frozen PBMCs for this part of our study. Thus, we additionally determined gamma-H2AX or 53BP1 foci in frozen PBMCs of 10 patients with CIS/early RRMS with CEL, 10 patients with CIS/early RRMS without CEL and 10 healthy controls. Demographics, clinical and MRI findings of these patients and controls are summarized in Table 2.

**Table 2:** Demographic and clinical data of patients and healthy controls of which previously frozen PBMCs were analyzed in this study.

	Patients without CEL	Patients with CEL	Healthy Controls	*P*-value
Number	10	10	10	n/a
CIS/early RRMS (number)	4/6	1/9	n/a	*p* = 0.30
Female/Male (number)	5/5	6/4	6/4	*p* = 0.81
Age, years, median (range)	32.3 (24–51)	29 (22–51)	28.5 (20–49)	*p* = 0.97
EDSS, median (range)	1.5 (0–5.5)	1.75 (0–3.5)	n/a	*p* = 0.67
T2w-lesions, number, median (range)	12 (2–32)	16 (7–79)	n/a	p = 0.12
CEL, number, median (range)	0	2 (1–3)	n/a	n/a
CEL, volume in cm^3^, median (range)	n/a	0 (0.02–0.38)	n/a	n/a
Immunomodulatory treatment, number				
None	3	7	n/a	*p* = 0.18
Glatiramer acetate	4	0	n/a	
Interferon-beta	2	3	n/a	
Dimethyl fumarate	1	0	n/a	

CIS = clinically isolated syndrome, RRMS = relapsing remitting multiple sclerosis, EDSS = expanded disability status scale, T2w = T2 weighted, CEL = contrast enhancing lesions, n/a = not applicable

In the majority of patients (18/20) MRI was performed on the day of blood withdrawal. The delay between blood withdrawal and MRI in the remaining 2 patients was 5 and 14 days. Patients with CEL had higher numbers of γ-H2AX foci per cell than patients without CEL (*p* = 0.03) and patients with CEL had a higher percentage of γ-H2AX positive cells (*p* = 0.03) than patients without CEL (Fig 3A and 3B). However, the γ-H2AX focus number per cell (*p* = 0.2) and the percentage of γ-H2AX positive cells (*p* = 0.63) did not differ between patients with CEL and healthy controls. Furthermore, a three-group comparison of patients with CEL, patients without CEL and healthy controls by Kruskal-Wallis test demonstrated no significant differences between the three groups in the number of γ-H2AX foci per cell (*p* = 0.09) as well as the percentage of γ-H2AX positive cells (*p* = 0.06). Neither the number of γ-H2AX foci per cell (r = 0.31; *p* = 0.39) nor the percentage of γ-H2AX positive cells (r = 0.15; *p* = 0.68) correlated with the number of CEL. Similarly, neither the number of γ-H2AX foci per cell (r = 0.16; *p* = 0.66) nor the percentage of γ-H2AX positive cells (r = 0.16; *p* = 0.66) correlated with the volume of CEL.

Neither the number of 53BP1 foci per cell (*p* = 0.91) nor the percentage of 53BP1 positive cells (*p* = 0.63) differed between patients with and without CEL (Fig 3C and 3D). Likewise, Kruskal-Wallis tests showed no significant differences of the number of 53BP1 foci per cell (*p* = 0.52) as well as the percentage of 53BP1 positive cells (*p* = 0.49) between patients with CEL, patients without CEL and healthy controls.

## Discussion

In this study, we analyzed the significance of γ-H2AX and 53BP1 foci in nuclei of PBMCs as potential diagnostic and disease activity markers for CIS/early RRMS using immunocytochemistry combined with fully automated immunofluorescence microscopy. The key findings of this work are an absence of any significant differences of γ-H2AX and 53BP1 foci levels between patients and healthy controls and no correlation of γ-H2AX and 53BP1 levels with MRI measures of MS and the EDSS in patients with CIS/early RRMS.

The median levels of γ-H2AX observed in our study, expressed as number of foci per cell (0.04), were similar to previously reported levels of γ-H2AX foci per cell in healthy subjects (0.05–0.1 foci/cell, see Valdiglesias et al. for review) [24,25]. Our data therefore confirm that there is a low background rate of DNA damage foci in isolated human PBMCs. Low levels of lymphocytic DNA damage foci and underlying DNA double-strand breaks may thus represent a physiological phenomenon, which likely has no harmful consequences as it is counteracted by DNA damage repair mechanisms [26]. As expected, lymphocytic γ-H2AX and 53BP1 foci were strongly correlated, consistent with the concept that generation of γ-H2AX foci in response to DNA double-strand breaks provides a platform for the recruitment of further DNA damage response proteins [10]. However, γ-H2AX and 53BP1 foci did not always colocalize (see Fig 1B), suggesting that γ-H2AX might not be a necessary prerequisite for recruitment of 53BP1 or that expression of 53BP1 exceeds the duration of γ-H2AX expression.

The absence of any significant differences of γ-H2AX or 53BP1 foci between patients with CIS/early RRMS and healthy controls suggests that lymphocytic DNA double-strand breaks are not associated with CIS/early MS and, therefore, appear unlikely to play a major role in the pathophysiology of MS. Our data are in contrast to previous findings by Grecchi et al., who reported a significantly (*p* = 0.025) higher percentage of γ-H2AX positive cells, as measured by immunofluorescence microscopy and manual evaluation of 500 cells per individual, in 19 patients with RRMS compared to 13 healthy controls, though there was a considerable overlap between both groups [17]. Among the reasons for the disparate findings may be the larger sample size of the current study as well as different patient characteristics with more patients in the earliest phase of MS in our work. Nevertheless, the similar EDSS values of the patients included in the two studies do not suggest major differences in clinical disability between the two patient populations. Although the study by Grecchi et al. included treatment naive patients only, the fact that some patients in our study were treated with immunomodulatory therapies does likewise not seem to be a confounder, as we did not observe any differences of γ-H2AX and 53BP1 in treated vs. untreated patients. The study by Grecchi et al. and our study used different methods for the fixation of cells. However, as in each study the method of fixation was identical for patients and controls, it appears unlikely that different fixation methods may explain the different results of both studies. While in contrast to the study by Grecchi et al. few of our patients did not undergo blood withdrawal and MRI on the same day, we do not feel that the short delay between blood withdrawal and MRI in these few patients might have distorted our results. It should be noted that the percentages of γ-H2AX positive cells reported by Grecchi et al. are higher than previously reported in PBMCs of healthy controls [25,27,28] and that the immunocytochemical staining of γ-H2AX positive cells shown in the work of Grecchi et al. suggests that cells with a pan-nuclear γ-H2AX staining, possibly representing apoptotic cells, were included in the analysis. Altogether, using a highly standardized and previously evaluated method for enumeration of discrete nuclear γ-H2AX foci, we could not observe an association of γ-H2AX foci with CIS/early RRMS, suggesting that γ-H2AX foci in PBMCs do not seem to be a promising diagnostic biomarker for CIS/early RRMS. Our results are consistent with the notion that MS patients have no generally increased risk of DNA-damage related diseases such as malignancies and, consequently, are unlikely to express elevated levels of DNA-damage related markers [29].

We found no correlation of γ-H2AX or 53BP1 foci with the number or volume of T2w lesions on cranial MRI, indicating that γ-H2AX or 53BP1 foci are not associated with these classical MRI markers of MS disease burden. Evaluation of γ-H2AX or 53BP1 foci in freshly isolated PBMCs with respect to CEL was limited by the fact that only two patients had CEL. Nevertheless, the very different γ-H2AX and 53BP1 foci levels in these two patients do not seem to suggest an association of γ-H2AX and 53BP1 foci with CEL.

To further address a possible association γ-H2AX and 53BP1 levels with CEL, we performed an additional analysis using previously frozen PBMCs of 10 patients with CIS/early RRMS with CEL, 10 patients with CIS/early RRMS without CEL and 10 healthy controls. Although, reminiscent of findings by Grecchi et al. [17], the number of γ-H2AX foci per cells as well as the percentage of γ-H2AX positive cells were higher in patients with as compared to patients without CEL, these data should be interpreted with great caution. Firstly, a two group comparison of γ-H2AX levels between patients with CEL and healthy controls and a three group comparison of γ-H2AX levels between patients with CEL, patients without CEL and healthy controls revealed no significant differences. Secondly, correction for multiple testing, e.g. by the Bonferroni method, would abolish the statistical significance of the differences between patients with and without CEL. Thirdly, there was no correlation between γ-H2AX levels and the number or volume of CEL. Fourthly, both the number of γ-H2AX foci per cell and the percentage of γ-H2AX positive cells completely overlapped between the three groups, questioning the clinical applicability and meaningfulness of γ-H2AX levels as a marker of disease activity. Altogether, although γ-H2AX levels appeared to be higher in two-group comparisons of patients with as compared to patients without CEL, our findings do not seem to suggest that γ-H2AX levels may be a promising disease activity marker for CIS/early RRMS.

Strengths of our study are the use of a standardized automated scoring method, minimizing observer influence and allowing for large numbers of scored cells per individual. Indeed, our automated reading system may also be useful to analyze other potential biomarkers in PBMCs of patients with MS with distinct subcellular staining patterns. Among the limitations of this work is the sample size. In particular, the small number of freshly isolated PBMCs from individuals with CEL precluded definitive conclusions on the association of γ-H2AX and 53BP1 levels in freshly isolated PBMCs with CEL.

In conclusion, the present study suggests that nuclear γ-H2AX and 53BP1 foci in PBMCs do not distinguish patients with CIS/early RRMS from healthy controls, arguing against a role of these parameters as diagnostic biomarkers for CIS/early MS. Furthermore, γ-H2AX and 53BP1 foci do not seem to be promising disease activity biomarkers in patients with CIS/early RRMS. Lymphocytic DNA double-strand breaks are thus unlikely to play a major role in the pathophysiology of MS.

## Supporting Information

S1 Table DNA damage parameters, demographic and clinical data of patients and healthy controls of which freshly isolated PBMCs were analyzed. Legend: EDSS: Expanded disability status scale; IMT: Immunomodulatory treatment; T2w: T2 weighted lesions; CEL: Contrast enhancing lesions; n/a: not applicable.
(XLSX)

S2 Table DNA damage parameters, demographic and clinical data of patients and healthy controls of which previously frozen PBMCs were analyzed. Legend: CEL: Contrast enhancing lesions; CEL-: CEL absent; CEL+: CEL present; EDSS: Expanded disability status scale; IMT: Immunomodulatory treatment; T2w: T2 weighted lesions; n/a: not applicable.
(XLSX)
